# Rectal Prolapse Associated with Extensive Anorectal Condyloma Acuminata

**DOI:** 10.4103/1319-3767.45064

**Published:** 2009-01

**Authors:** Hanan M. AlGhamdi, Shyam A. Parashar, Saleem Kawaja, Mona H. Ismail, Zeead M. AlGhamdi

**Affiliations:** King Fahad Hospital of the University, Department of Surgery, AlKhobar, Saudi Arabia; 1 King Fahad Hospital of the University, Department of Internal Medicine, AlKhobar, Saudi Arabia. E-mail: hananghamdi@yahoo.com

Sir,

Complete rectal prolapse (full-thickness and circumferential intussusception of the entire rectal wall through the anal canal) is not a common condition.[1] Furthermore, the presence of anorectal giant condyloma acuminata (Con A; a sexually transmitted disease caused by human papilloma viruses [HPV]) has been rarely reported in association with or as the probable cause of rectal prolapse.[2] We report a patient with extensive longstanding anorectal Con A with recurrent rectal prolapse that became irreducible and required urgent surgery for reduction and perianal and abdominal rectopexy.

**Figure 1:**
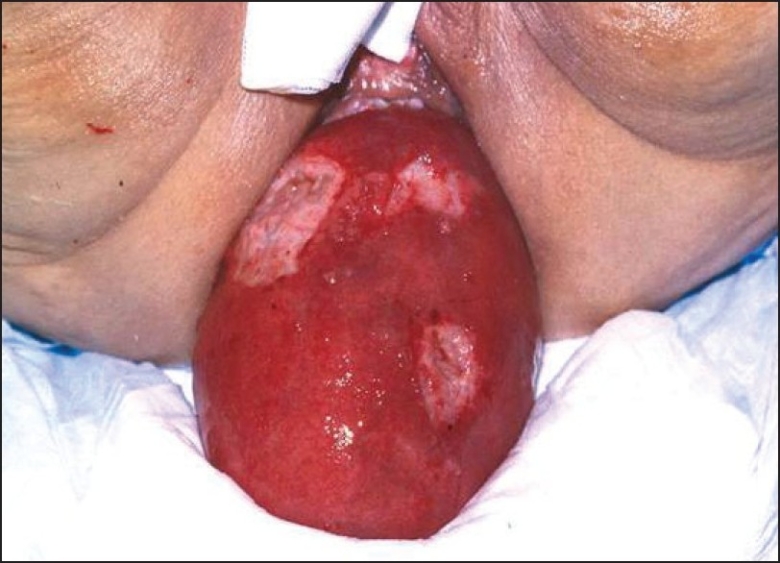
Rectal prolapse with inflammation, thickening, edematous, ulcerating, and anal condyloma acuminata

A 51-year-old woman, mother of six children, presented with longstanding history of extensive perineal and vaginal Con A for 15 years, which has been proved by microbiology, immunology, and histological examination to be due to human papilloma virus (HPV) with occasional concomitant candidal infection. She received different regimens of local and systemic medical treatment for the Con A and candida, but the perianal Con A persisted. She had a history of recurrent mild rectal bleeding that became heavier in the last year and also reported tensemus and occasional reducible anal mass on straining. She developed complete rectal prolapse on straining [Figure 1]. Under general anesthesia, the edematous rectum could only be reduced through lower abdominal laparotomy. The grossly patulous anus was repaired by using Thiersch's procedure, and then rectosigmoidopexy was done. The perianal condyloma was fulgurated by diathermy. The tissue histology was consistent with Con A with no malignancy.

The precise cause of rectal prolapse is not fully defined. However, a number of associated abnormalities and conditions have been described. Nearly 50% of rectal prolapse is caused by longstanding constipation with chronic straining. Other causes include increased intraabdominal pressure, a deep pouch of Douglas, weakness of the pelvic floor, decreased resting anal sphincter pressure, neurological disorders, and some parasitic infections (amebiasis and schistosomiasis).[1]

On the other hand, Con A (warts) is generally a sexually transmitted disease of adults caused by HPV usually type 6 and 11 double-stranded DNA viruses that replicate in squamous epithelial cells. It occurs mainly in genital and perianal areas (moist area) but rarely in the rectum. The lesion may progress into large papillomatous proliferations called condylomata gigantean or Buschke-Lowenstein tumor that penetrates deep into the underlying tissue, usually without metastatic potential.[3,4]

This case report demonstrates some of the challenges in caring for patients with Con A and the consequences. The importance of aggressive extirpation therapy was not appreciated until very late in her course. The presence of anorectal giant Con A and the association with rectal prolapse is rarely recognized in the literature.
